# Supplementary material for: Functional characterization of specialized immune cells in a cnidarian reveals an ancestral antiviral program
Source: Nat Commun. 2026 Apr 24;17:5699. doi: 10.1038/s41467-026-72325-8 (PMC13319465; doi:10.1038/s41467-026-72325-8)
Supplement: Supplementary file 1 — Supplementary Information [file 41467_2026_72325_MOESM1_ESM.pdf]

Functional characterization of specialized immune cells in a cnidarian reveals an ancestral antiviral program

Itamar Kozlovski[1,9*], Ton Sharoni[1], Shani Levy[2,3], Adrian Jaimes-Becerra[1], Shani Talice[4,5], Hee-Jin Kwak[1], Daria Aleshkina[1], Reuven Aharoni[1], Xavier Grau-Bové[2], Ola Karmi[6], Benyamin Rosental[4], Arnau Sebe-Pedros[2,7,8], Yehu Moran[1*].

[1]Department of Ecology, Evolution and Behavior, The Alexander Silberman Institute of Life Sciences, Faculty of Science, The Hebrew University of Jerusalem, Jerusalem, Israel

[2]Centre for Genomic Regulation (CRG), Barcelona Institute of Science and Technology (BIST), Barcelona, Spain

[3]Department of Blue Biotechnologies and Sustainable Mariculture, Leon H. Charney School of Marine Sciences, University of Haifa, Haifa, Israel

[4]The Shraga Segal Department of Microbiology, Immunology, and Genetics, Faculty of Health Sciences, Center for Regenerative Medicine and Stem Cells, Ben Gurion University of the Negev, Beer Sheva, Israel

[5]The Goldman Sonnenfeldt School of Sustainability and Climate Change, Ben-Gurion University of the Negev, Beer Sheva, Israel

[6]Research Infrastructure Facility, Alexander Silberman Institute of Life Sciences, Faculty of Science, The Hebrew University of Jerusalem, Jerusalem, Israel

[7]Universitat Pompeu Fabra (UPF), Barcelona, Spain

[8]ICREA, Barcelona, Spain

[9]Present address: Department of Tissue Dynamics and Regeneration, Max Planck Institute for Multidisciplinary Sciences, Göttingen, Germany

*Corresponding authors: itamar.kozlovski@mail.huji.ac.il (IK); yehu.moran@mail.huji.ac.il (YM)

This PDF file includes:

Supplementary Figures S1 to S10

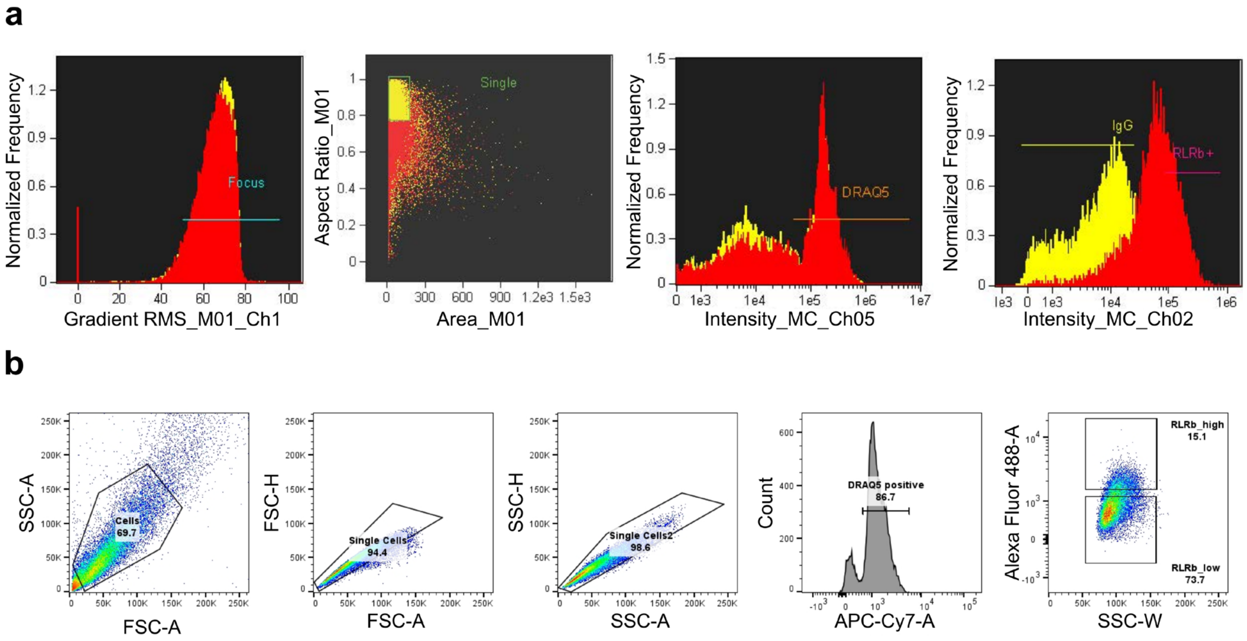

Supplementary Fig. 1. Gating strategies of intracellularly immunostained cells. (a) ImageStream gating strategy. Focused cells were gated based high gradient RMS, aggregates and debris were excluded based on aspect ratio and manual inspection of individual cell images, intact cells were selected by the nuclear marker DRAQ5, RLRb signal was defined relative to IgG control. **(b)** Gating strategy for flow cytometry.

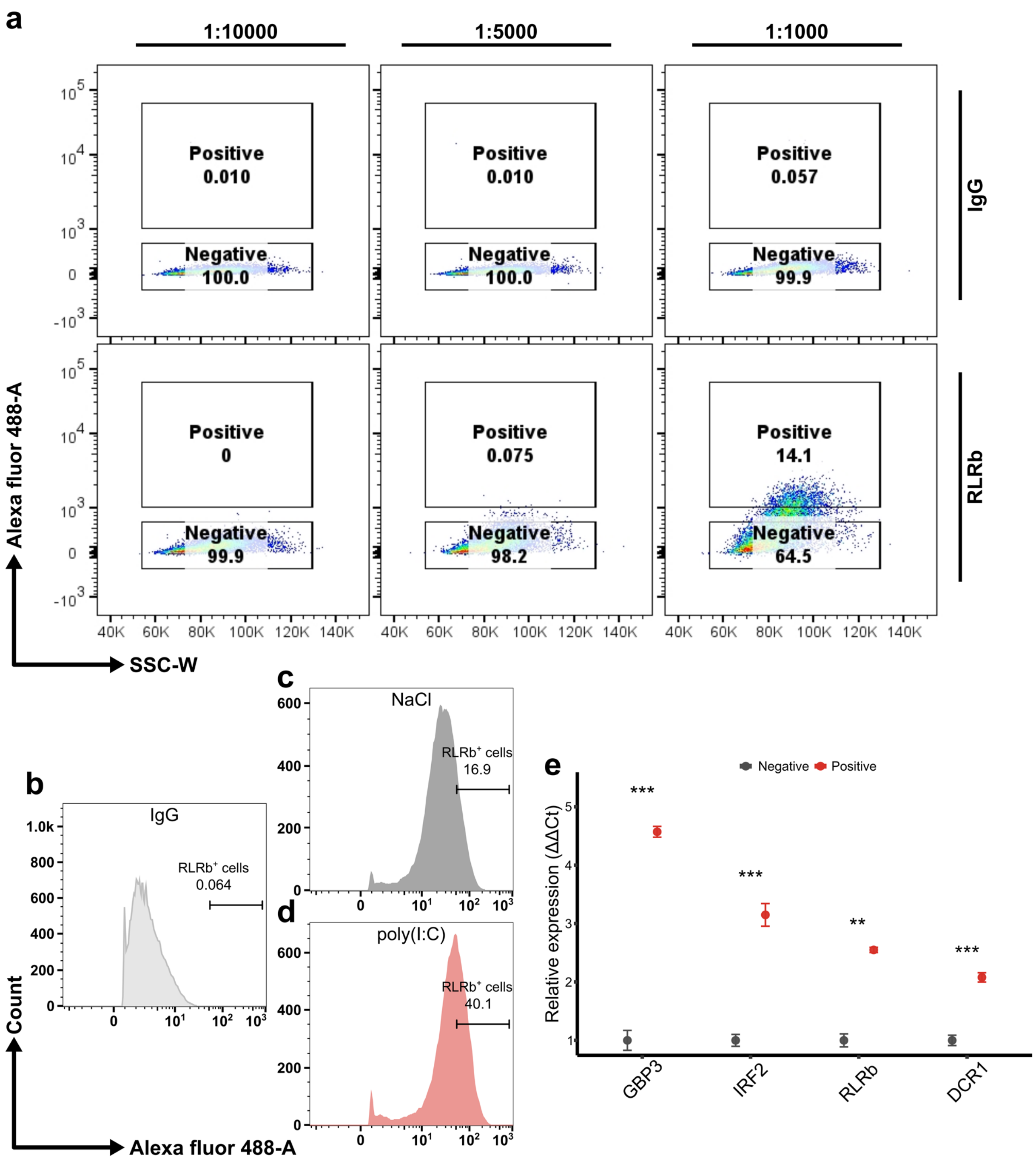

Supplementary Fig. 2. Validation of intracellular immunostaining for single-cell detection of RLRb
(a) Titration of the RLRb antibody relative to IgG control. **(b)** flow cytometry results of IgG stained control, **(c)** NaCl control and **(d)** poly(I:C) injected cells. **(e)** RT-qPCR validation of immune gene enrichment in RLRb$^+$ cells. FACS-isolated RLRb$^+$ (Positive) and RLRb$^-$ (Negative) cells were analyzed for expression of GBP3, IRF2, RLRb, and DCR1. RLRb$^+$ cells show significantly higher relative expression (ΔΔCt) of all four genes compared with RLRb$^-$ cells ($P < 0.01$-0.001). Error bars represent mean ± SEM of 3 biological replicates. Source data are provided as a Source Data file.

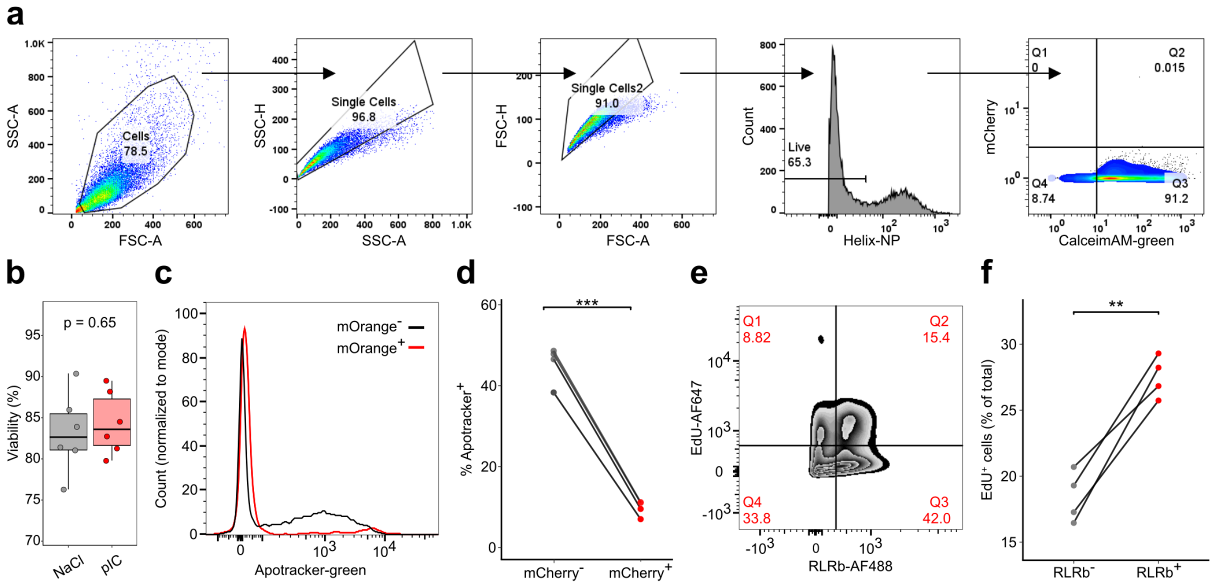

Supplementary Fig. 3. Apoptosis is reduced and proliferation is increased in RLRb^{+} cells. (a) Single cells were gated as shown and viability was determined using helix-NP and CalceinAM green. (b) Viability of dissociated *N. vectensis* cells. Live (Calcein-AM Green^{+}, Sytox Blue^{-}) cells were quantified after dissociation and resuspension in PBS-containing buffer. Cell viability was comparable between NaCl- and pIC-treated animals (~80-85%; two-sided t-test, p = 0.65). Boxplots show median, interquartile range, and individual biological replicates. **(c)** Representative histogram of Apotracker-green fluorescence showing reduced apoptosis in mCherry^{+} (RLRb^{+}) cells (red) compared to mCherry^{-} cells (black). mCherry^{+} cells display a clear left-shift in Apotracker signal, indicating fewer apoptotic cells. **(d)** Quantification of Apotracker^{+} cells across biological replicates (n = 4). The percentage of apoptotic cells was significantly lower in the mCherry^{+} population compared to the mCherry^{-} fraction (*p < 0.001, paired t-test). **(e)** Representative flow cytometry density plot showing EdU incorporation (AF647) versus RLRb labeling (AF488). Quadrants indicate RLRb^{+}/EdU^{+} double-positive cells (Q2), RLRb^{-}/EdU^{+} cells (Q1), and corresponding negative fractions. **(f)** Quantification of EdU^{+} cells across replicates (n = 4). RLRb^{+} cells exhibited a significantly higher proportion of EdU-positive cells compared to RLRb^{-} cells (p < 0.01, paired t-test), indicating increased proliferation. Source data are provided as a Source Data file.

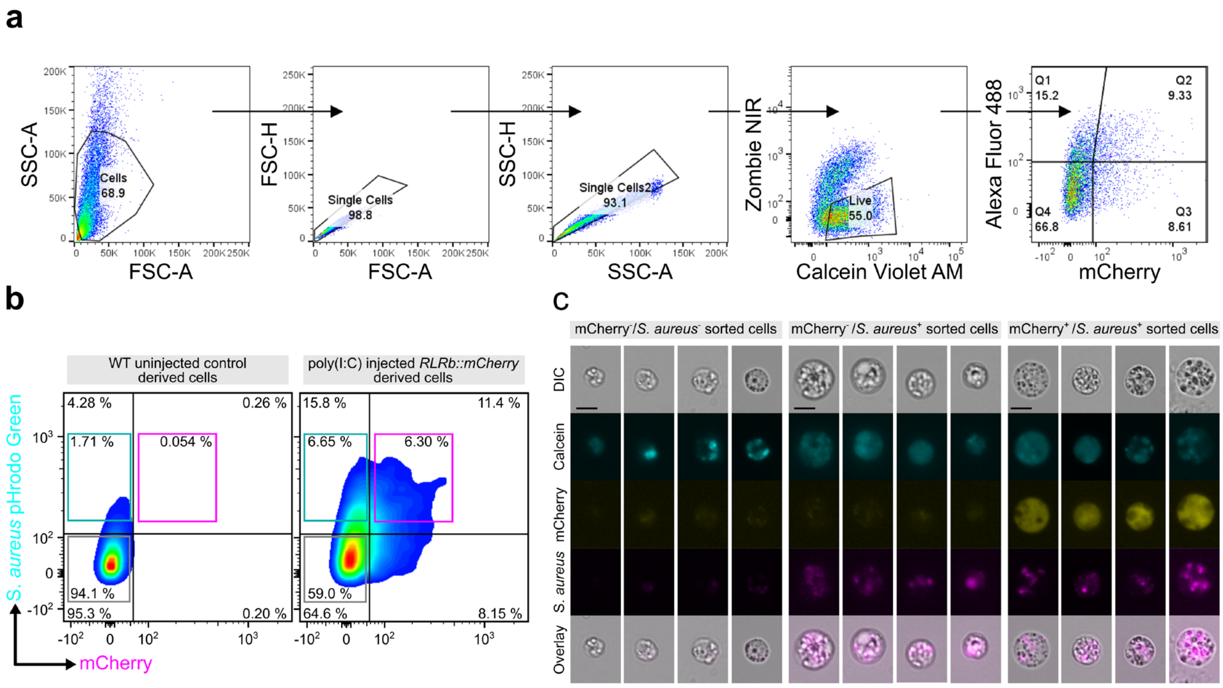

Supplementary Fig. 4. Flow cytometry gating strategy and microscopy validation of phagocytosis. (a) Single cells were gated as before. Calcein violet AM and Zombie NIR were used to determine viability. mCherry was plotted against the phagocytic probe and gates were defined with respect to WT untreated controls. **(b)** Representative flow cytometry density plots showing uptake of pHrodo™ Green-conjugated *Staphylococcus aureus* bioparticles versus mCherry fluorescence in dissociated cells from wild-type uninjected controls and poly(I:C)-injected *RLRb::mCherry* animals. Percentages indicate the proportion of cells in each gated quadrant. **(c)** Representative DIC and fluorescence images of FACS-sorted mCherry^{-}/*S. aureus*$^{-}$, mCherry^{-}/*S. aureus*$^{+}$, and mCherry^{+}/*S. aureus*$^{+}$ cells. Calcein marks live cells. Scale bar, 10 μm.

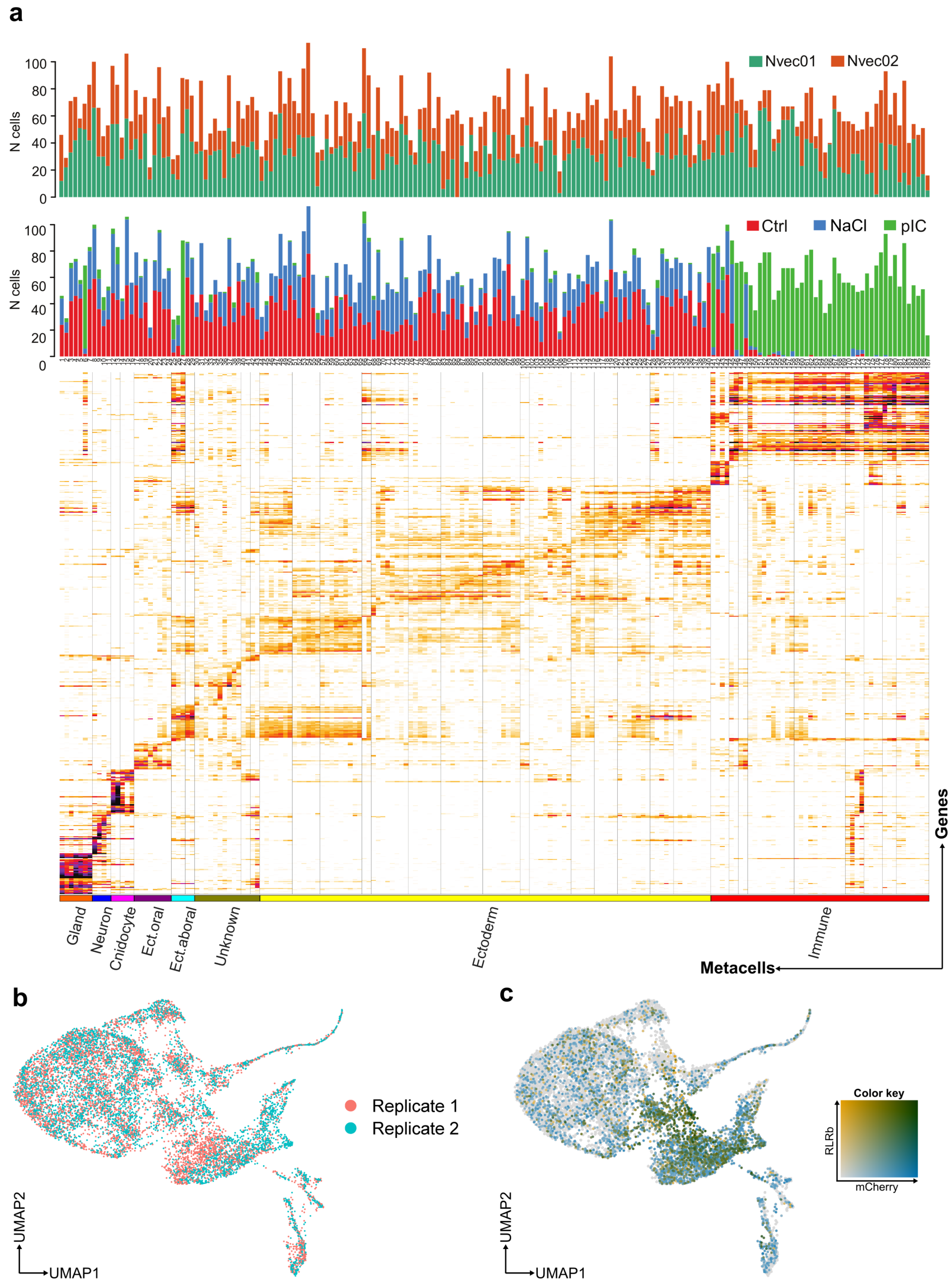

Supplementary Fig. 5. Metacell and scRNAseq are reproducible and reveal poly(I:C) responsive cell states. (a) Top panel shows the distribution of metacells across two biological replicates. Bottom panel shows the distribution of metacells across conditions. The heatmap shows gene expression (rows) across metacells (columns). **(b)** scRNAseq distribution of cells across two biological replicates visualized on a UMAP projection. **(c)** Co-expression of mCherry and RLRb visualized by additive color blending. Single cells are projected onto a UMAP embedding and colored by scaled expression of mCherry (blue) and RLRb (orange). Low expression of both genes is shown in grey, whereas intermediate hues indicate

co-expression proportional to the relative contribution of each gene. Increased color intensity reflects higher overall expression.

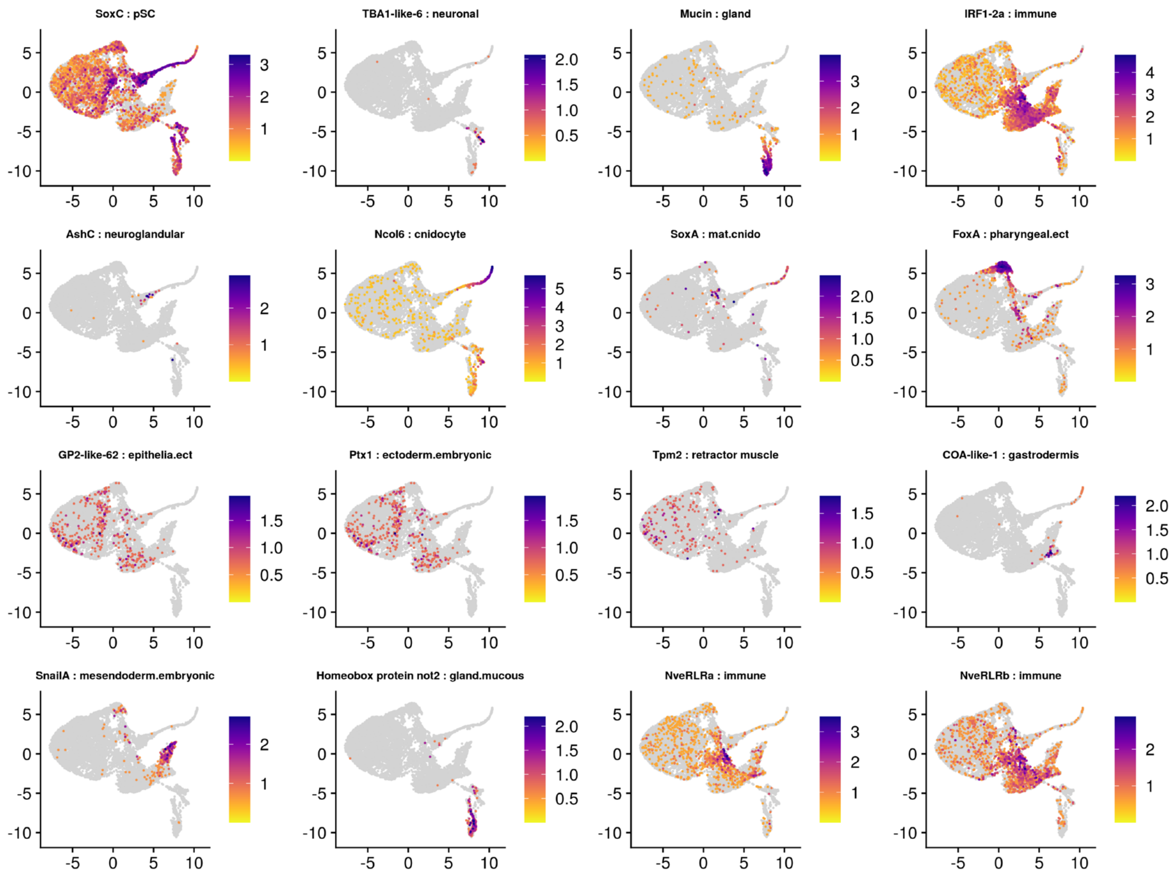

Supplementary Fig. 6. Cell type specific markers. (a) Expression of cell type specific markers reported by Cole *et.al* [1] is shown on UMAP projections. The marker and the cell type or tissue of origin are indicated.

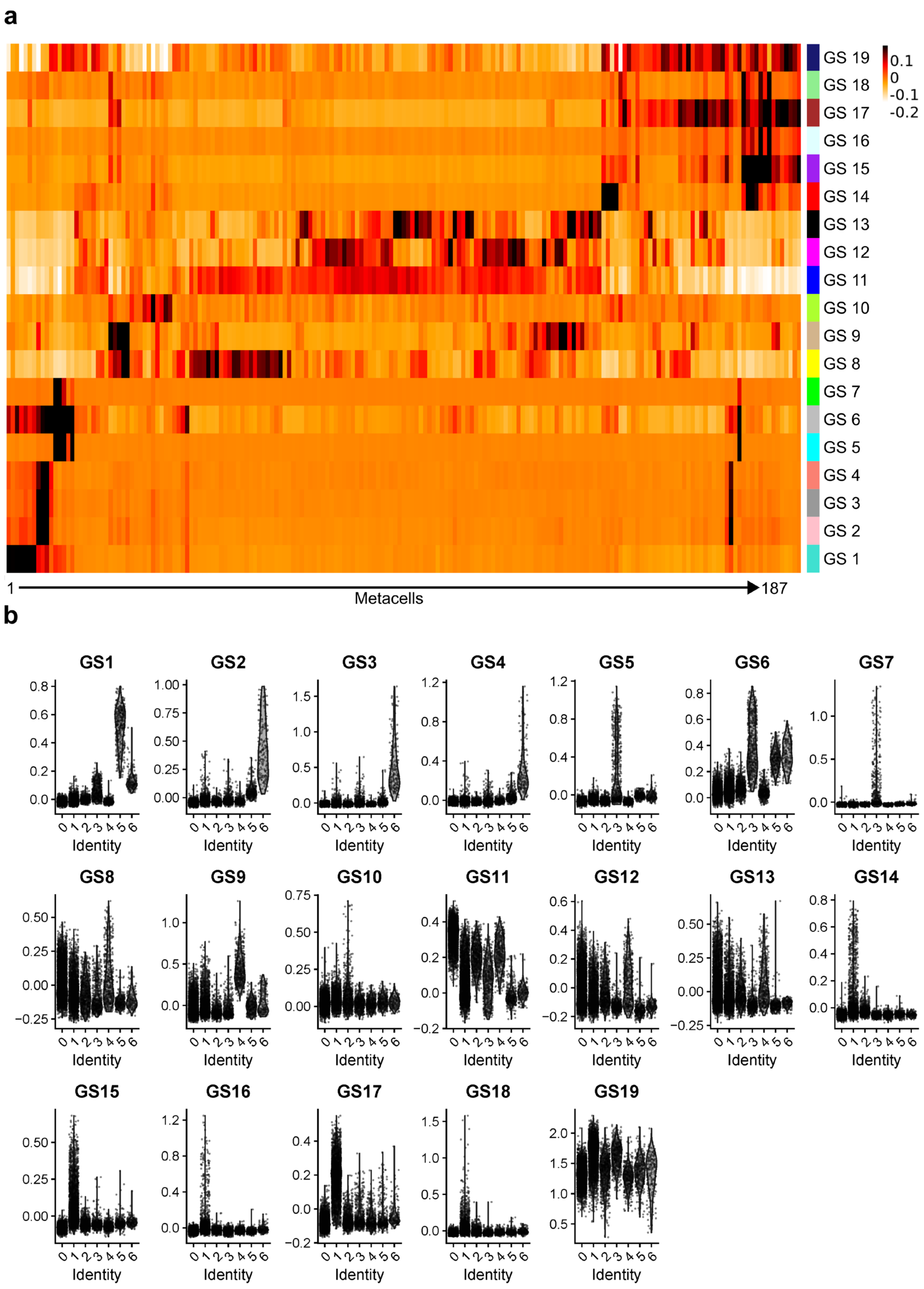

Supplementary Fig. 7. Identification of cell-type specific gene modules. (a) WGCNA analysis revealed 19 distinct gene modules (named GS 1-19) across metacells. The heatmap shows the enrichment of the modules across metacells. **(b)** Average expression score per module was computed across cell clusters

and visualized as violin plots.

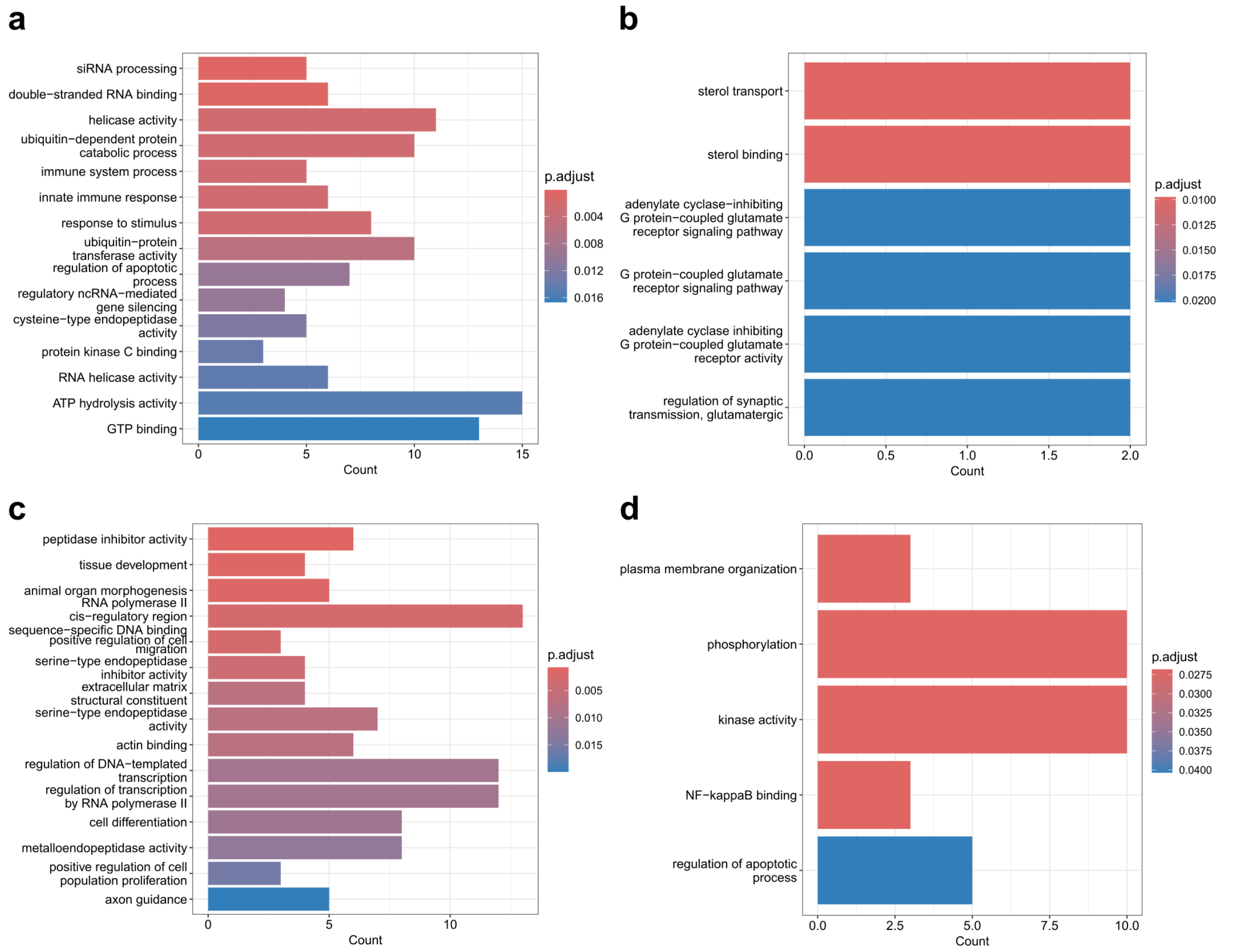

Supplementary Fig. 8. Over-representation analysis (ORA) of immune specific gene modules. (a) ORA of GS17, **(b)** GS16, **(c)** GS14, **(d)** GS15. Genes found in each gene module were used for analysis. Each of the gene modules was enriched in the immune cluster. Top terms are shown.

a

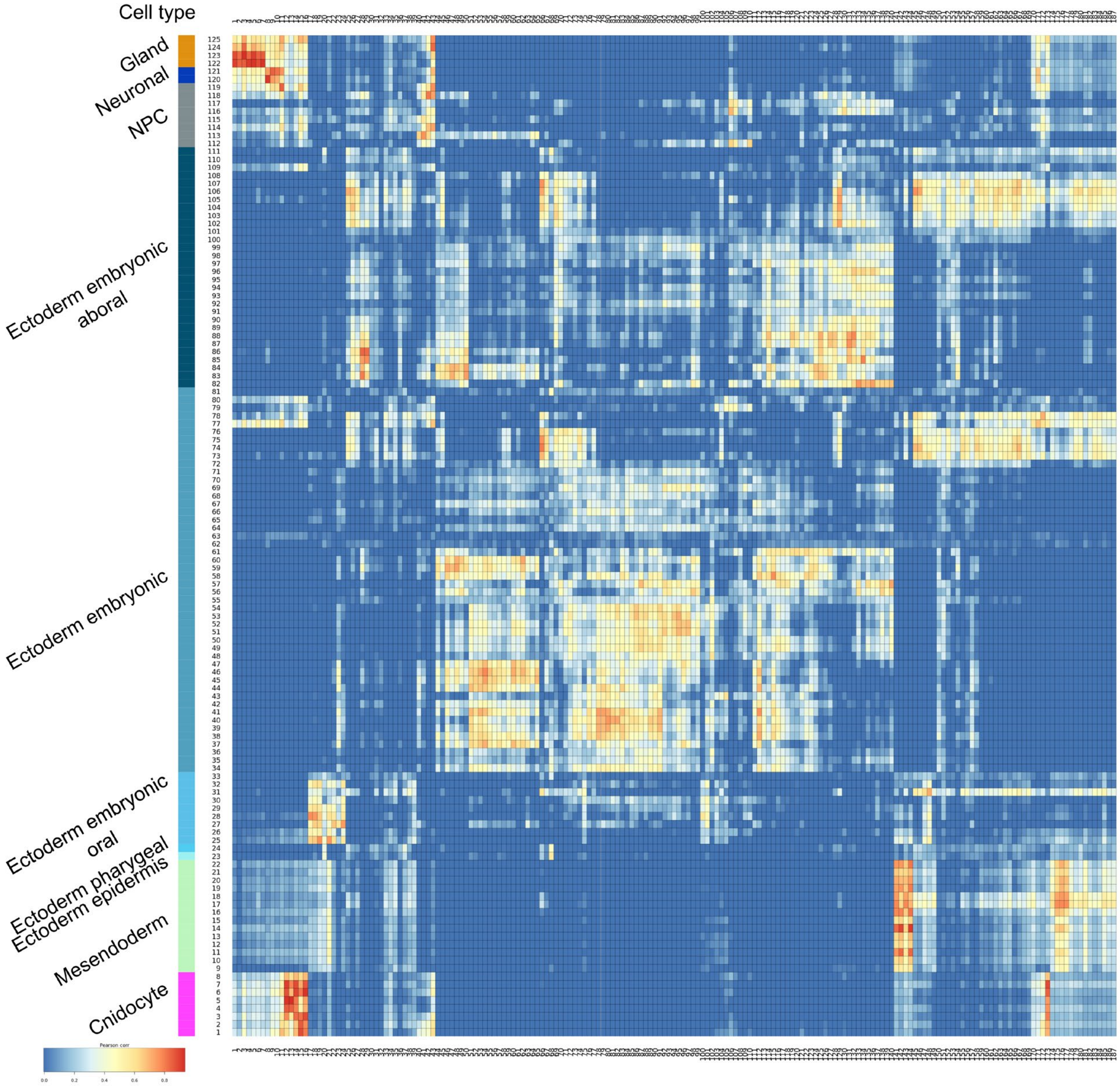

Supplementary Fig. 9. Correlation of metacells found in this study and metacells identified by Cole *et.al* [1]. (a) Metacells identified by cole *et al.* (n=125) were labelled according to cell-types (rows), metacells found in this study (n=187) are shown as columns. Pearson correlation coefficient is shown, with red indicating high correlation and blue indicating low correlation.

a

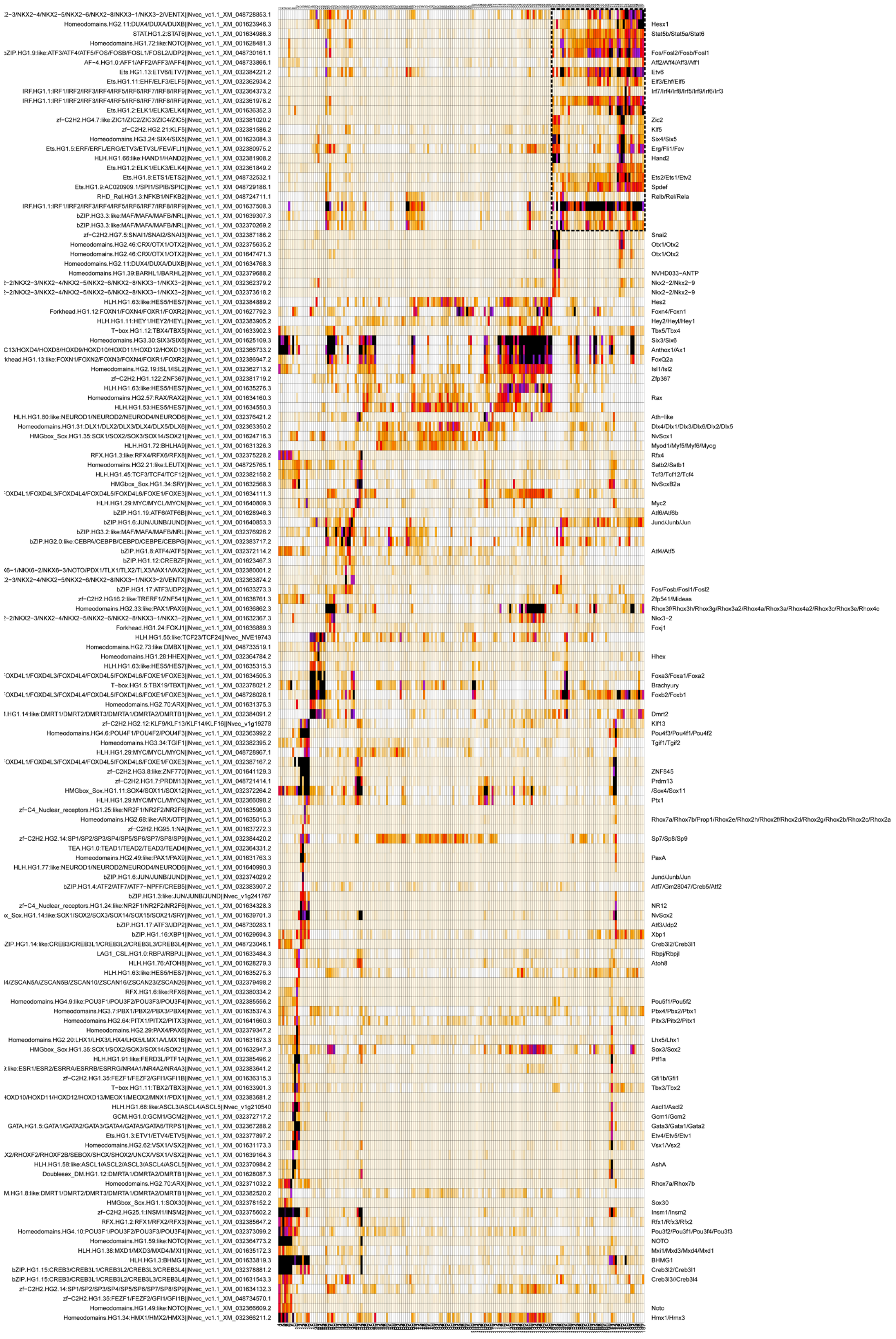

Supplementary Fig. 10. Transcription factors expression per metacell. (a) Heatmap showing transcription factors expression across metacells. The dashed rectangle represents metacells and transcription factors that were enriched upon poly(I:C) treatment.

References

1 Cole, A. G. *et al.* Updated single cell reference atlas for the starlet anemone Nematostella vectensis. *Frontiers in Zoology* **21**, 8 (2024).
