# Supplementary material for: Functional characterization of specialized immune cells in a cnidarian reveals an ancestral antiviral program
Source: Nat Commun. 2026 Apr 24;17:5699. doi: 10.1038/s41467-026-72325-8 (PMC13319465; doi:10.1038/s41467-026-72325-8)
Supplement: Supplementary file 2 — Description of Additional Supplementary Files [file 41467_2026_72325_MOESM2_ESM.pdf]

Functional characterization of specialized immune cells in a cnidarian reveals an ancestral antiviral program

Itamar Kozlovski[1,9*], Ton Sharoni[1], Shani Levy[2,3], Adrian Jaimes-Becerra[1], Shani Talice[4,5], Hee-Jin Kwak[1], Daria Aleshkina[1], Reuven Aharoni[1], Xavier Grau-Bové[2], Ola Karmi[6], Benyamin Rosental[4], Arnau Sebe-Pedros[2,7,8], Yehu Moran[1*].

[1]Department of Ecology, Evolution and Behavior, The Alexander Silberman Institute of Life Sciences, Faculty of Science, The Hebrew University of Jerusalem, Jerusalem, Israel

[2]Centre for Genomic Regulation (CRG), Barcelona Institute of Science and Technology (BIST), Barcelona, Spain

[3]Department of Blue Biotechnologies and Sustainable Mariculture, Leon H. Charney School of Marine Sciences, University of Haifa, Haifa, Israel

[4]The Shraga Segal Department of Microbiology, Immunology, and Genetics, Faculty of Health Sciences, Center for Regenerative Medicine and Stem Cells, Ben Gurion University of the Negev, Beer Sheva, Israel

[5]The Goldman Sonnenfeldt School of Sustainability and Climate Change, Ben-Gurion University of the Negev, Beer Sheva, Israel

[6]Research Infrastructure Facility, Alexander Silberman Institute of Life Sciences, Faculty of Science, The Hebrew University of Jerusalem, Jerusalem, Israel

[7]Universitat Pompeu Fabra (UPF), Barcelona, Spain

[8]ICREA, Barcelona, Spain

[9]Present address: Department of Tissue Dynamics and Regeneration, Max Planck Institute for Multidisciplinary Sciences, Göttingen, Germany

*Corresponding authors: itamar.kozlovski@mail.huji.ac.il (IK); yehu.moran@mail.huji.ac.il (YM)

This PDF file includes:

Legends for Supplementary Data S1-S25

Supplementary Data legends

Supplementary Data S1. Raw ImageStream features for intracellular RLRb and IgG control staining used in Fig. 1. Raw single-cell quantitative features exported from IDEAS 6.3 software following imaging flow cytometry analysis of intracellularly stained cells labeled with anti-RLRb or anti-IgG control antibodies. The table contains six sheets, each corresponding to an independent biological replicate. Rows represent individual acquired objects (single cells), indexed by object number. Columns include morphological, intensity, texture, granularity, circularity, localization, and fluorescence-based measurements across acquisition channels, including bright field (Ch01), Alexa Fluor 488 (Ch02, 488 nm laser), and Alexa Fluor 647 (Ch05, 642 nm laser), as well as derived features such as area, aspect ratio, circularity, gradient RMS, modulation, contrast, and bright detail statistics. Data were acquired using an Amnis ImageStreamX Mk II (Luminex) with a 60× objective under low-flow, high-sensitivity conditions. Focused single cells were gated based on Gradient RMS and area–aspect ratio parameters in Ch01, and all downstream analyses were performed on this population. These raw features were used for quantitative comparisons, statistical testing, and dimensionality reduction analyses shown in Fig. 1.

Supplementary Data S2. DESeq2 results for RLRb-high versus RLRb-low cells. Differential expression analysis corresponding to Fig. 1g–i was performed using DESeq2 with the default two-sided Wald test and Benjamini-Hochberg adjustment for multiple comparisons. Columns show baseMean, log2FoldChange (RLRb-high vs RLRb-low), lfcSE, stat, pvalue, padj, and protein/domain annotations.

Supplementary Data S3. Gene set enrichment analysis of RLRb-high versus RLRb-low cells. Gene set enrichment analysis corresponding to Fig. 1i was performed using clusterProfiler with the fgsea algorithm on genes ranked by differential expression between RLRb-high and RLRb-low cells. P values were adjusted for multiple comparisons using the Benjamini–Hochberg method. The table reports enrichment statistics for each gene set, including the normalized enrichment score (NES), nominal p value, and adjusted p value.

Supplementary Data S4. DESeq2 results for mCherry-positive versus mCherry-negative cells. Differential expression analysis corresponding to **Fig. 3g–i** was performed using DESeq2 with the default two-sided Wald test, and p values were adjusted for multiple comparisons using the Benjamini-Hochberg method. Columns report standard DESeq2 statistics and gene annotations.

Supplementary Data S5. Gene set enrichment analysis of mCherry-positive versus mCherry-negative cells. Gene set enrichment analysis results corresponding to Fig. 3j, performed using clusterProfiler[1] on genes ranked by differential expression between mCherry-positive and mCherry-negative cells. **The table reports enrichment statistics for each gene set, including normalized enrichment score, nominal p value, and adjusted p value.**

Supplementary Data S6. Single-cell RNA-seq quality metrics and metacell expression matrix. Sheet “QC_metrics” contains CellRanger run summary statistics for the two biological replicates (Nvec01 and Nvec02), including cell numbers, sequencing depth, mapping rates, and RNA capture metrics. Sheet “Metacell_UMIs” contains normalized UMI fractions for each gene across all metacells and was used to generate the metacell-level expression profiles shown in Fig. 5b.

Supplementary Data S7. Differential expression analysis of poly(I:C)- versus NaCl-treated embryos by scRNA-seq. Differential expression analysis was performed on single-cell RNA-seq data using Seurat with MAST (test.use = "MAST"), which applies a hurdle model for differential expression testing. P values were adjusted for multiple comparisons using the Bonferroni method, as reported by Seurat in p_val_adj. Columns report MAST/Seurat statistics (p_val, avg_log2FC, pct.1, pct.2, p_val_adj) and protein/domain annotations.

Supplementary Data S8. Marker genes reported by Cole *et al.* [2]. List of cell type-associated marker genes curated from Cole *et al.*[2], including cell type or state assignment, marker gene name, description, corresponding *Nematostella* gene identifiers (NVE), and DToL transcript IDs.

Supplementary Data S9. Cell cluster marker genes identified in this study. Marker genes defining cell clusters were identified using Seurat FindMarkers with the default Wilcoxon rank-sum test and a minimum log fold-change threshold of 0.25. P values were adjusted for multiple comparisons using the

Bonferroni method, as reported by Seurat in p_val_adj. These markers were used for cluster annotation and visualization in Supplementary Fig. 6.

Supplementary Data S10. MAST differential expression analysis of cluster 1 versus all other clusters. Differential expression analysis comparing cells in cluster 1 with all other clusters was performed using Seurat FindMarkers with MAST, which applies a hurdle model for differential expression testing. P values were adjusted for multiple comparisons using the Bonferroni method, as reported by Seurat in p_val_adj.

Supplementary Data S11. WGCNA gene module assignment. Results of weighted gene co-expression network analysis (WGCNA)[3] used to identify gene modules across metacells. The table lists gene identifiers, assigned module, module membership score, predicted Pfam domain architecture, and best reciprocal BLAST hit (homo_bbh).

Supplementary Data S12. Over-representation analysis of the GS17 (brown) gene module. Over-representation analysis results for genes in the GS17 (brown) module, performed using clusterProfiler[1]. Enrichment significance was assessed using a hypergeometric test, and p values were adjusted for multiple comparisons using the Benjamini-Hochberg method.

Supplementary Data S13. Over-representation analysis of the GS16 (lightcyan) gene module. Over-representation analysis results for genes in the GS16 (lightcyan) module, performed using clusterProfiler[1]. Enrichment significance was assessed using a hypergeometric test, and p values were adjusted for multiple comparisons using the Benjamini-Hochberg method.

Supplementary Data S14. Over-representation analysis of the GS14 (red) gene module. Over-representation analysis results for genes in the GS14 (red) module, performed using clusterProfiler[1]. Enrichment significance was assessed using a hypergeometric test, and p values were adjusted for multiple comparisons using the Benjamini-Hochberg method.

Supplementary Data S15. Over-representation analysis of the GS15 (purple) gene module. Over-representation analysis results for genes in the GS15 (purple) module, performed using clusterProfiler[1]. Enrichment significance was assessed using a hypergeometric test, and p values were adjusted for multiple comparisons using the Benjamini-Hochberg method.

Supplementary Data S16. Marker genes identified in sub-clusters 0-3. Marker genes for sub-clusters 0-3 identified using Seurat[4] *FindMarkers* with the default Wilcoxon rank-sum test, a minimum log fold-change threshold of 0.25, and Bonferroni-adjusted p values as reported by Seurat. These markers were used for visualization and analysis in Fig. 6.

Supplementary Data S17. Over-representation analysis of sub-cluster 0. Over-representation analysis results for genes in sub-cluster 0, performed using clusterProfiler[1]. Enrichment significance was assessed using a hypergeometric test, and p values were adjusted for multiple comparisons using the Benjamini-Hochberg method.

Supplementary Data S18. Over-representation analysis of sub-cluster 1. Over-representation analysis results for genes in sub-cluster 1, performed using clusterProfiler[1]. Enrichment significance was assessed using a hypergeometric test, and p values were adjusted for multiple comparisons using the Benjamini-Hochberg method.

Supplementary Data S19. Over-representation analysis of sub-cluster 2. Over-representation analysis results for genes in sub-cluster 2, performed using clusterProfiler[1]. Enrichment significance was assessed using a hypergeometric test, and p values were adjusted for multiple comparisons using the Benjamini-Hochberg method.

Supplementary Data S20. Over-representation analysis of sub-cluster 3. Over-representation analysis results for genes in sub-cluster 3, performed using clusterProfiler[1]. Enrichment significance was assessed using a hypergeometric test, and p values were adjusted for multiple comparisons using the Benjamini-Hochberg method.

Supplementary Data S21. Orthogroups identified by OrthoFinder. Tab-separated OrthoFinder[5] output used for orthogroup identification in **Fig. 7**. Columns list orthogroup IDs and the corresponding gene or protein identifiers from *Nematostella vectensis* (Nvec) and *Stylophora pistillata* (Spi). *N. vectensis* genes

derive from poly(I:C)-stimulated immune transcriptomic experiments, including cluster 1 (immune cells), and *S. pistillata* genes derive from cGAMP-stimulated immune activation datasets[6].

Supplementary Data S22. Shared single-copy orthogroups and gene expression used for correlation analysis. Table of shared single-copy orthogroups between *Nematostella vectensis* and *Stylophora pistillata*, including gene-level differential expression statistics, gene and protein annotations, and orthogroup assignments. Differential expression analysis for the N. *vectensis* data was performed using Seurat FindMarkers with MAST (test.use = "MAST"), comparing cluster 1 (immune) cells versus all other clusters. P values were adjusted for multiple comparisons using the Bonferroni method, as reported by Seurat in p_val_adj .S. *pistillata* data derive from cGAMP-stimulated immune activation experiments[6]. These data were used for cross-species correlation analysis in **Fig. 7c**.

Supplementary Data S23. Shared genes used for cross-species correlation analysis. List of shared genes with adjusted *p* value < 0.05 used for correlation analysis in Fig. 7e. The table integrates gene-level differential expression statistics from cluster 1 (immune) *Nematostella vectensis* cells, computed using Seurat *FindMarkers* with MAST and Bonferroni-adjusted p values, with corresponding expression metrics from publicly available cGAMP-stimulated *Nematostella* data analyzed using DESeq2 with the default two-sided Wald test and Benjamini-Hochberg-adjusted p values[7]. Columns report DESeq2- and MAST-derived statistics, protein and domain annotations, and identifiers used for correlation analysis.

Supplementary Data S24. Genes shared between cluster 1 in non-injected controls and adult immune cells. Marker genes for cluster 1 in the non-injected control condition were identified using Seurat *FindMarkers* with the default Wilcoxon rank-sum test (min.pct = 0.25, logfc.threshold = 0.25) and Bonferroni-adjusted p values. The table lists genes shared with the adult immune cell cluster identified by Cole et al.[2], together with differential expression statistics and protein/domain annotations.

Supplementary Data S25. Gene model conversion dictionary and annotations. Dictionary linking gene models used in this study to corresponding *Nematostella vectensis* NVE and NV2 gene models. Columns include DToL gene model identifiers[8], NVE and NV2 mappings, gene names, predicted protein homologs, and Pfam[9] domain annotations.

References

1 Wu, T. *et al.* clusterProfiler 4.0: A universal enrichment tool for interpreting omics data. *Innovation (Camb)* **2**, 100141 (2021). https://doi.org/10.1016/j.xinn.2021.100141

2 Cole, A. G. *et al.* Updated single cell reference atlas for the starlet anemone Nematostella vectensis. *Frontiers in Zoology* **21**, 8 (2024).

3 Langfelder, P. & Horvath, S. WGCNA: an R package for weighted correlation network analysis. *BMC bioinformatics* **9**, 1-13 (2008).

4 Satija, R., Farrell, J. A., Gennert, D., Schier, A. F. & Regev, A. Spatial reconstruction of single-cell gene expression data. *Nature biotechnology* **33**, 495-502 (2015).

5 Emms, D. M. & Kelly, S. OrthoFinder: phylogenetic orthology inference for comparative genomics. *Genome biology* **20**, 1-14 (2019).

6 Li, Y. *et al.* cGLRs are a diverse family of pattern recognition receptors in innate immunity. *Cell* **186**, 3261-3276 e3220 (2023). https://doi.org/10.1016/j.cell.2023.05.038

7 Margolis, S. R. *et al.* The cyclic dinucleotide 2' 3'-cGAMP induces a broad antibacterial and antiviral response in the sea anemone Nematostella vectensis. *Proceedings of the National Academy of Sciences* **118**, e2109022118 (2021).

8 Fletcher, C. *et al*. The genome sequence of the starlet sea anemone, Nematostella vectensis (Stephenson, 1935). *Wellcome Open Research* **8**, 79 (2023).

9 Finn, R. D. *et al*. Pfam: the protein families database. *Nucleic acids research* **42**, D222-D230 (2014).
