# Supplementary material for: Functional characterization of specialized immune cells in a cnidarian reveals an ancestral antiviral program
Source: Nat Commun. 2026 Apr 24;17:5699. doi: 10.1038/s41467-026-72325-8 (PMC13319465; doi:10.1038/s41467-026-72325-8)
Supplement: Supplementary file 5 — Transparent Peer Review file [file 41467_2026_72325_MOESM5_ESM.pdf]

nature portfolio

# Peer Review File

# Functional characterization of specialized immune cells in a cnidarian reveals an ancestral antiviral program

Corresponding Author: Professor Yehu Moran

**This file contains all reviewer reports in order by version, followed by all author rebuttals in order by version.**

Version 0:

Reviewer comments:

Reviewer #1

(Remarks to the Author)
This is a well-conducted study in which authors were able to identify a cell type subset, present in Nematostella, responsible for the antiviral immune response in embryos. As already demonstrated, the viral dsRNA is recognized by RLRb receptors. Here authors correlate the localization and the gene expression on RLRb+ cells after microinjection of poli(I:C) in transgenic mCherry::RLRb Nematostella model. Evidence were obtained by different approaches such as flow cytometry, morphological stain using antibody against RLRb and bioinformatics comparative analysis between their scRefSeq in embryo with transcriptome obtained from the adult by Cole et.al or from cGAMP treated Nematostella polyps published by Margolis et.al or in the coral S. pistillata, confirming the existence of a pool of ancestor molecule and genes involved in antiviral response that belong to morphologically distinct immune cells population.
All the methods employed are adequately described and correct with appropriate negative controls carried out. Results are presented clearly and language adopted is substantially correct.
I do not have many comments except for the confusing use of words “marker genes”. An example is on Pag. 3 line 6 “lack of cell type specific markers” which seems contradictory with what was stated 5 lines below “enabling the identification of cell type-specific marker genes (29, 30)”. Another example is on page 7 “Next we annotated cell types using previously published cell type markers (40) (table S8). Using these markers, we were able to identify gland cells, neurons, embryonic ectodermal cells, and a small subset of nematocytes (Fig. 5F, fig. S6A and table S9). The expression of IRF1-2a, which was reported as a putative immune cell marker (40)”. Since it seems that marker genes exist, and some of those are showed up-regulated in GSEA for differentially expressed genes analysis, I was wondering if it is possible to validate the scRNAseq results by qPCR.
Pag 10 “However, studies of the innate immune system in invertebrates are scarce and confounded by a small phyletic sampling, primarily limited to flies and nematodes (64).” I would suggest softening this sentence that refers to antiviral immunity instead of innate immunity
One of the main problems I had when performing single cell sorting is that cells (from coelomic fluid or in the hemolymph) are extremely “unhappy” when withdrawn outside from the animals. Morphology changes quickly and often I need to use anticoagulant to prevent clotting. Do the authors experience this kind of problem? The morphology (and vitality) gets even worse when the cells are washed or suspended in PBS which I notice was employed in several technique. Is it possible to add somewhere a sentence in which authors state that PBS (or PBS modified to have a correct osmolarity) does not affect the morphology and the vitality of the cells?

Reviewer #2

(Remarks to the Author)
The authors analyze cellular immune response in the planula larva of the starlet sea anemone, Nematostella vectensis. They use the PRR RLRb as a marker for immune activity. They visualize RLRb using both immunofluorescence and a transgenic Nematostella line carrying nCherry under the control of an RLRb regulatory region. They analyze characteristics of RLRb+ cells from dissociated 2-day old planulae that are WT, mock injected, or injected with poly(I:C). They compare transcriptome compositions of RLRb positive and negative cells. The describe the distinct morphology of RLRb+ cells and the upregulation of other innate immune regulators. They visualize RLRb cells in intact planula larvae after exposure to poly(I:C) and compare cell features of induced RLRb cells using scRNAseq on dissociated planula cells. They use Pseudotime prediction to trace gene expression as the cells emerge and identify a proliferative stage in the expansion of

these cells. Finally, they compare the RLRb cells to similar studies in adult Nematostella polyps and in the coral Stylophora pistillata to identify similar cells different developmental stages and in a divergent anthozoan.

The manuscript addresses an important topic that has long been of interest to comparative immunologists. The planula cells that respond to immune challenge appear to activate a suite of genes and potentially proliferate. They appear to have counterparts in distantly related cnidarians.

A main question is what these changes mean in terms of cell type identity (i.e., whether these cells represent a “cell state of hyper innate immune activation” or a genuine stably differentiated “cell type.” The Discussion methodically addresses this issue, but it seems that there are other ways to contribute to this determination. Information of the fate and of these RLRb cells after immune stimulation has receded may lend insight. Do the RLTb cells remain differentiated? Do they dedifferentiate? Do they apoptose?

In phagocytosis assays, better imaging may be useful to further characterize the cells morphology and behavior beyond what is evident from FACs and whole organism images. For example, are the cells motile? Time lapse microscopy following these cells during phagocytosis may lend some insight into any particulars of morphology or behavior.

Overall, the manuscript is quite detailed and should be of broad interest. More insight into the question of immune cell differentiation vs more widespread cellular innate immune activation would improve the paper.

Specific points for consideration:

1. What is the fate of the increased number of RLRb+ planula cells after metamorphosis into the polyp? Given that this study identified a number of markers for these cells beyond RLRb, why were these cells not visualized in the adult polyp also?

2. What are the RLRb negative phagocytic cells? Are they specialized also or have they just not yet upregulated RLRb?

3. Many of the gene names in the text seem vertebrate specific. Can the authors discuss issues of gene orthology?

4. Page 10 Discussion end of Paragraph 1: There are actually a lot of studies of innate immunity in invertebrates outside of flies and C. elegans. Should broaden this statement or qualify the type of information that is limited.

5. Page 11, middle: Cells with macrophage- and neutrophil-like character may be ancient but is is unclear whether these represent ancient cell types or convergent cell phenotypes. Maybe qualify this statement.

Minor issues:
Page 2 line 1 abstract: replace reconstructing with reconstruct

Page 33, legend, Fig.3I. Wrong reference number for Lewandowska?

Fig 5C: gene names are impossible to read. Maybe show in another way here and expand the volcano plot in the Supplementary figs?

Page numbers and line numbers would be useful in the review process.

Reviewer #3

(Remarks to the Author)
This manuscript addresses the evolutionary origin of immune cells by identifying and functionally characterizing a distinct population of cells activated by the viral mimic poly(I:C) in embryos of the cnidarian Nematostella vectensis. This study represents a substantial step forward and provides an absolute treasure trove of information and data on this topic that will help propel our understanding of how innate immunity evolved. The manuscript was a pleasure to read, well-organized and clear, and each section flowed logically from the previous one and the results were convincing. The approaches, including the innovative tools and creative experimental designs, are going to set a new standard for performing this type of study addressing similar questions. These tools include both standard flow cytometry and imaging flow cytometry, immunoprecipitation + mass spec for assessing antibody specificity, bulk RNAseq, single-cell RNAseq, a transgenic reporter line, and a phagocytosis assay assessed by flow cytometry. Each of these approaches provides evidence and data to support the authors' ability to characterize the target cell population and support their hypothesis that this is a novel population of specialized immune cells involved in antiviral immunity. I appreciated the detail provided, including the gating strategies for all flow cytometry experiments.

I have two major critiques that must be addressed:

1. The authors make it clear throughout that they chose to focus on embryos of Nematostella, which is fine. They even provide some text in the discussion about how their results with embryos compare with results from previous studies by Cole et al. on adults and Margolis et al. on 4 week old polyps. However the authors need to provide a clear rationale for focusing on embryos. Was it simply that some of their approaches would only work well in embryos, or were there other reasons for this decision? Including a few more sentences on the rationale for choosing embryos and also a focus on the limitations of

focusing on embryos (for making comparative claims) is warranted in the Introduction and again in the Discussion to tie it up.

2. Looking at the Data availability section, I feel what the authors have provided is very minimal, and woefully inadequate. If this is going to be a model or a new standard for how studies of novel cell types are characterized, as I hope it can be, then there is a huge amount of supplemental information/data that should be provided so others can replicate or follow what was done and validate the results or apply it to their own study. This is essential for others to be able to utilize the same approaches and analysis methods in other systems. I do appreciate the FigShare links that were provided, and I have looked through them. I also appreciate all of the supplemental figures & tables provided. However, what is missing is a supplemental methods text explanation of these supplemental figures & tables. For an example, please see Cazet et al. 2023 Genome Res. 33: 283-298. Supplemental_Material.pdf file for a guide for what needs to be included.

Also, it does seem that only minimal information has been provided for the single-cell RNAseq experiments and libraries. At a bare minimum, a table of statistics showing how many cells were loaded, how many were captured, and then the other summary statistics that are provided by CellRanger including fraction of reads per cell, mean reads per cell, median genes per cell, total genes detected, reads mapped to genome, et cet, must be provided. It is impossible to assess data quality without this information.

What also really needs to be provided in addition to these, is step-by-step descriptions of ALL computational analyses conducted as part of this study, including all relevant code, formatted both as markdown and HTML documents. This could be deposited in GitHub or a similar repository. For an example of a study where this was organized and executed quite well, again please see Cazet et al. 2023 Genome Res. 33: 283-298. Please follow this standard and provide similar descriptions of all computational analyses for each section of the manuscript.

I also see that Table 1 shows the ImageStream raw data. This is not adequate in terms of flow cytometry data that needs to be provided. Please see the Current Protocols chapter "Preparing a Minimum Information about a Flow Cytometry Experiment (MIFlowCyt) Compliant Manuscript Using the International Society for Advancement of Cytometry (ISAC) FCS File Repository (FlowRepository.org)"
https://currentprotocols.onlinelibrary.wiley.com/doi/full/10.1002/0471142956.cy1018s61
At a minimum, all of the raw .fcs files for ALL flow cytometry experiments must be deposited into the FlowRepository.

Once all of this Data availability has been provided and updated, I will need to re-evaluate everything to make sure it was done to the necessary standard, that the data quality is high enough, and that the analyses were appropriate. As it stands, I am unable to evaluate the manuscript at this level. I have evaluated some other manuscripts that made an inadequate effort to provide code for all analyses in a study in a single text document for all experiments and it was completely impossible to follow & unacceptable for publication.

Beyond that, this study is a very impressive contribution and I personally look forward to further digging into the specific genes and pathways that were uncovered as there is a massive amount of cool science to follow up on. I also look forward to future studies performed in adults as that will be super interesting and an important next step to the story.

Version 1:

Reviewer comments:

Reviewer #1

(Remarks to the Author)
The authors responded point by point to this reviewer's questions. The paper has been substantially improved with the addition of new figures (supplementary S2, S4 and S5), and I am fully satisfied with the detailed explanations that have been included in the new version of the paper.

Reviewer #2

(Remarks to the Author)
The authors have responded to my reviewer questions with changes to the manuscript or explanations to resolve the questions that I had. The study addresses fundamental problems of animal immunity and will likely be of wide interest to immunologists and evolutionary biologists.

Reviewer #3

(Remarks to the Author)
I am now satisfied with the updated version of this manuscript and with the authors' response to my review comments. I appreciate all the data that has now been provided, including the Supplemental Material, the QC information on the single-cell experiments, the GitHub repository, and the Zenodo repository. I was able to access all files in the repositories, download and open them, and verify that everything was properly uploaded and accessible, and appeared to be correct. I also appreciate that there is now clarity about why the authors chose to use early developmental stages for their study, and that the manuscript now includes an explanation of this reasoning.

Kudos to the authors on the updated manuscript. I am very much looking forward to seeing this published and to seeing the impact it will have.

**Open Access** This Peer Review File is licensed under a Creative Commons Attribution 4.0 International License, which permits use, sharing, adaptation, distribution and reproduction in any medium or format, as long as you give appropriate credit to the original author(s) and the source, provide a link to the Creative Commons license, and indicate if changes were made.
In cases where reviewers are anonymous, credit should be given to 'Anonymous Referee' and the source.
The images or other third party material in this Peer Review File are included in the article's Creative Commons license, unless indicated otherwise in a credit line to the material. If material is not included in the article's Creative Commons license and your intended use is not permitted by statutory regulation or exceeds the permitted use, you will need to obtain permission directly from the copyright holder.
To view a copy of this license, visit https://creativecommons.org/licenses/by/4.0/

## REVIEWER COMMENTS

We would like to thank all the reviewers for their thoughtful comments and constructive feedback, which have significantly improved the quality of our manuscript.

**Reviewer #1 (Remarks to the Author):**

This is a well-conducted study in which authors were able to identify a cell type subset, present in Nematostella, responsible for the antiviral immune response in embryos. As already demonstrated, the viral dsRNA is recognized by RLRb receptors. Here authors correlate the localization and the gene expression on RLRb+ cells after microinjection of poli(I:C) in transgenic mCherry::RLRb Nematostella model. Evidence were obtained by different approaches such as flow cytometry, morphological stain using antibody against RLRb and bioinformatics comparative analysis between their scRefSeq in embryo with transcriptome obtained from the adult by Cole et.al[1] or from cGAMP treated Nematostella polyps published by Margolis et.al[2] or in the coral S. pistillata, confirming the existence of a pool of ancestor molecule and genes involved in antiviral response that belong to morphologically distinct immune cells population.
All the methods employed are adequately described and correct with appropriate negative controls carried out. Results are presented clearly and language adopted is substantially correct.
I do not have many comments except for the confusing use of words “marker genes”. An example is on Pag. 3 line 6 “lack of cell type specific markers” which seems contradictory with what was stated 5 lines below “enabling the identification of cell type-specific marker genes (29, 30)”. Another example is on page 7 “Next we annotated cell types using previously published cell type markers (40) (table S8). Using these markers, we were able to identify gland cells, neurons, embryonic ectodermal cells, and a small subset of nematocytes (Fig. 5F, fig. S6A and table S9). The expression of IRF1-2a, which was reported as a putative immune cell marker (40)”. Since it seems that marker genes exist, and some of those are showed up-regulated in GSEA for differentially expressed genes analysis, I was wondering if it is possible to validate the scRNAseq results by qPCR.
Pag 10 “However, studies of the innate immune system in invertebrates are scarce and confounded by a small phyletic sampling, primarily limited to flies and nematodes (64).” I would suggest softening this sentence that refers to antiviral immunity instead of innate immunity
One of the main problems I had when performing single cell sorting is that cells (from coelomic fluid or in the hemolymph) are extremely “unhappy” when withdrawn outside from the animals. Morphology changes quickly and often I need to use anticoagulant to prevent clotting. Do the authors experience this kind of problem? The morphology (and vitality) gets even worse when the cells are washed or suspended in PBS which I notice

was employed in several technique. Is it possible to add somewhere a sentence in which authors state that PBS (or PBS modified to have a correct osmolarity) does not affect the morphology and the vitality of the cells?

I do not have many comments except for the confusing use of words “marker genes”. An example is on Pag. 3 line 6 “lack of cell type specific markers” which seems contradictory with what was stated 5 lines below “enabling the identification of cell type-specific marker genes (29, 30)”. Another example is on page 7 “Next we annotated cell types using previously published cell type markers (40) (table S8). Using these markers, we were able to identify gland cells, neurons, embryonic ectodermal cells, and a small subset of nematocytes (Fig. 5F, fig. S6A and table S9). The expression of IRF1-2a, which was reported as a putative immune cell marker (40)”. Since it seems that marker genes exist, and some of those are showed up-regulated in GSEA for differentially expressed genes analysis, I was wondering if it is possible to validate the scRNAseq results by qPCR.

In the revised manuscript, we removed the phrase “lack of cell-type–specific markers” to avoid confusion. As noted, the study by Cole *et al.* indeed identified (or more correctly, suggested) cell-type–specific markers, and these are in strong agreement with our single-cell analysis. This concordance is demonstrated in Figure S9, which shows the Pearson correlation between the two datasets and further supports our clustering. Additionally, we present the expression of selected marker genes from Cole *et al.* in our dataset in Figure S6 and used these markers to guide our cluster annotations, as shown in Figure 5F.

Regarding qPCR validation, we have now added additional results in Figure S2E demonstrating the enrichment of the immune genes *RLRb*, *GBP3*, *IRF2*, and *DCR1* in $RLRb^+$ ($RLRb^{high}$) cells isolated by intracellular immunostaining followed by FACS. Together with the strong concordance with the published dataset of Cole *et al.* and the high reproducibility observed in our single-cell sequencing results (Figures S5A-S5B; see response to Reviewer 3), these data provide robust support for our clustering and marker gene assignments.

We do not expect that additional bulk qPCR validation would provide further insight, as bulk RT-qPCR lacks the cellular resolution of single-cell RNA-seq. By averaging expression across heterogeneous cell populations, bulk qPCR can mask or even invert differences that are specific to particular cell subsets or driven by changes in cell-type composition rather than per-cell expression. Consequently, we believe that additional qPCR assays would not meaningfully strengthen the validation already established by our single-cell data and additional experimental confirmation.

Pag 10 “However, studies of the innate immune system in invertebrates are scarce and confounded by a small phyletic sampling, primarily limited to flies and nematodes (64).” I would suggest softening this sentence that refers to antiviral immunity instead of innate immunity

Thanks. It now reads: “However, our understanding of antiviral immune responses in invertebrates remains incomplete, in part because most studies have focused on a few model systems such as flies and nematodes[3].”

One of the main problems I had when performing single cell sorting is that cells (from coelomic fluid or in the hemolymph) are extremely “unhappy” when withdrawn outside from the animals. Morphology changes quickly and often I need to use anticoagulant to prevent clotting. Do the authors experience this kind of problem? The morphology (and vitality) gets even worse when the cells are washed or suspended in PBS which I notice was employed in several technique. Is it possible to add somewhere a sentence in which authors state that PBS (or PBS modified to have a correct osmolarity) does not affect the morphology and the vitality of the cells?

We thank the reviewer for raising this important point. In our hands, dissociated *Nematostella vectensis* cells remain morphologically intact and viable under the PBS-based conditions used for FACS and downstream assays. To directly address this concern, we quantified cell viability after dissociation and resuspension in PBS containing 2 % BSA using a viability dye. As shown in the new Figure S3B, viability remained high (~80–85 %) and was not significantly different between NaCl and pIC-treated animals (unpaired two-tailed t-test, $p = 0.65$), indicating that our buffer conditions, including PBS osmolarity, do not compromise cell survival on the timescale of our experiments.

From an ecological and physiological perspective, *N. vectensis* is an estuarine species that naturally experiences and tolerates large fluctuations in salinity, with reported survival across ~9–52 ppt and successful maintenance in the laboratory over a broad salinity range[4-6].

This broad tolerance likely explains why transient exposure to PBS-based buffers is well tolerated by its cells. Consistent with this, multiple studies, including ours, using *Nematostella* embryos, larvae, and adults routinely employ PBS (often with BSA) for washes, immunostaining, and cell-based assays without reporting adverse effects on morphology or viability[1,7,8].

This is now stated in the *Methods* section: “Dissociated cells tolerated PBS-based buffers well and retained high viability (> 80 %) and normal morphology during all procedures, consistent with previous reports successfully using PBS for live cell assays and dissociation in *Nematostella*[1,8].”

**Reviewer #2 (Remarks to the Author):**

The authors analyze cellular immune response in the planula larva of the starlet sea anemone, Nematostella vectensis. They use the PRR RLRb as a marker for immune activity. They visualize RLRb using both immunofluorescence and a transgenic Nematostella line carrying mCherry under the control of an RLRb regulatory region. They analyze characteristics of RLRb+ cells from dissociated 2-day old planulae that are WT, mock injected, or injected with poly(I:C). They compare transcriptome compositions of RLRb positive and negative cells. They describe the distinct morphology of RLRb+ cells and the upregulation of other innate immune regulators. They visualize RLRb cells in intact planula larvae after exposure to poly(I:C) and compare cell features of induced RLRb cells using scRNAseq on dissociated planula cells. They use Pseudotime prediction to trace gene expression as the cells emerge and identify a proliferative stage in the expansion of these cells. Finally, they compare the RLRb cells to similar studies in adult Nematostella polyps and in the coral Stylophora pistillata to identify similar cells different developmental stages and in a divergent anthozoan.

The manuscript addresses an important topic that has long been of interest to comparative immunologists. The planula cells that respond to immune challenge appear to activate a suite of genes and potentially proliferate. They appear to have counterparts in distantly related cnidarians.

A main question is what these changes mean in terms of cell type identity (i.e., whether these cells represent a “cell state of hyper innate immune activation” or a genuine stably differentiated “cell type.” The Discussion methodically addresses this issue, but it seems that there are other ways to contribute to this determination. Information of the fate and of these RLRb cells after immune stimulation has receded may lend insight. Do the RLTb cells remain differentiated? Do they dedifferentiate? Do they apoptose?

In phagocytosis assays, better imaging may be useful to further characterize the cells morphology and behavior beyond what is evident from FACs and whole organism images. For example, are the cells motile? Time lapse microscopy following these cells during phagocytosis may lend some insight into any particulars of morphology or behavior.

Overall, the manuscript is quite detailed and should be of broad interest. More insight into the question of immune cell differentiation vs more widespread cellular innate immune activation would improve the paper.

Specific points for consideration:

1. What is the fate of the increased number of RLRb+ planula cells after metamorphosis into the polyp? Given that this study identified a number of markers for these cells beyond RLRb, why were these cells not visualized in the adult polyp also?

2. What are the RLRb negative phagocytic cells? Are they specialized also or have they just not yet upregulated RLRb?

3. Many of the gene names in the text seem vertebrate specific. Can the authors discuss issues of gene orthology?

4. Page 10 Discussion end of Paragraph 1: There are actually a lot of studies of innate immunity in invertebrates outside of flies and C. elegans. Should broaden this statement or qualify the type of information that is limited.

5. Page 11, middle: Cells with macrophage- and neutrophil-like character may be ancient but it is unclear whether these represent ancient cell types or convergent cell phenotypes. Maybe qualify this statement.

Minor issues:

Page 2 line 1 abstract: replace reconstructing with reconstruct

Page 33, legend, Fig.3I. Wrong reference number for Lewandowska?

Fig 5C: gene names are impossible to read. Maybe show in another way here and expand the volcano plot in the Supplementary figs?

Page numbers and line numbers would be useful in the review process.

**We thank the reviewer for the constructive feedback. In the revised manuscript, we have included new data to address the reviewers' comments. We believe this new information improves the manuscript and provides experimental data to support our single cell sequencing analysis.**

A main question is what these changes mean in terms of cell type identity (i.e., whether these cells represent a "cell state of hyper innate immune activation" or a genuine stably differentiated "cell type." The Discussion methodically addresses this issue, but it seems that there are other ways to contribute to this determination. Information of the fate and of these RLRb cells after immune stimulation has receded may lend insight. Do the RLRb cells remain differentiated? Do they dedifferentiate? Do they apoptose?

To further address the reviewer's question regarding the fate of $RLRb^+$ cells after immune stimulation, we performed apoptosis and proliferation assays. We found that **RLRb::mCherry$^+$ cells show significantly reduced apoptosis and increased proliferation compared to mCherry $^-$ cells** (Supplementary Fig. 3c–S3f), indicating that these cells are preferentially maintained and expanded following poly(I:C) treatment. This provides a mechanistic explanation for the increased abundance of the immune cluster (Fig. 6a).

Regarding differentiation, our transcriptomic analyses revealed **enrichment of differentiation-related gene modules** within the immune cluster upon stimulation (Fig. 7f and Supplementary Fig. 8c), suggesting that activation may promote differentiation processes. However, as noted in the Discussion, **direct experimental evidence for differentiation (e.g., lineage tracing)** is beyond the scope of this study.

Importantly, multiple lines of evidence already support the interpretation that the immune cluster represents a bona fide **cell type**, including (i) the strong overlap between embryonic immune cells and the adult immune cells described by Cole *et al.* (31/48 markers shared), (ii) consistent transcriptional responses across developmental stages, (iii) distinct morphology of $RLRb^+$ cells, (iv) the absence of markers of other known cell types, (v) the negligible viral load in embryos, and (vi) pseudotime trajectories indicating expansion of a pre-existing $RLRb^+$ population. As discussed, these observations do not exclude the possibility that additional cell types transiently activate immune programs; both processes may occur simultaneously.

Together, our new apoptosis/proliferation data and the transcriptomic and morphological evidence presented in the manuscript support the view that the $RLRb^+$ population reflects a stable immune cell type present at basal conditions and expanded upon immune activation.

In phagocytosis assays, better imaging may be useful to further characterize the cells morphology and behavior beyond what is evident from FACs and whole organism images. For example, are the cells motile? Time lapse microscopy following these cells during phagocytosis may lend some insight into any particulars of morphology or behavior.

We performed additional morphological analyses of phagocytic cells and now provide fluorescence microscopy images of the sorted phagocytic and non-phagocytic fractions to complement the flow cytometry data in Fig. 4 (see Supplementary Fig. 4). In agreement with our flow and imaging cytometry of mCherry positive and RLRb high cells, cells that were positive to mCherry and engulfed the *S .aureus* pHrodo Green bioparticle were large and granular compared to mCherry negative cells.

With respect to the suggestion of time-lapse microscopy, we believe this approach would not yield biologically meaningful insight in our system for several reasons: (i) the phagocytosis assay can only be performed on **dissociated cells**, as intact embryos do not respond to the fixed-bacteria probe, (ii) dissociated embryonic cell suspensions are **dominated by highly motile ciliated cells**, which makes reliable tracking of other cell types technically unfeasible, and (iii) the motility of isolated embryonic cells under imaging conditions is **unlikely to reflect their in vivo behavior**, rendering time-lapse observations difficult to interpret (in our experience with *Nematostella*, most cilia are used to generate water flow while the cells are integrated in the intact tissue, yet, they start moving due to cilia beating as soon as the tissue is dissociated). For these reasons, we consider the current combination of flow cytometry and fluorescence microscopy to provide the most robust characterization of phagocytic cell morphology available within the constraints of this assay.

Regarding cell motility, we found evidence at the transcriptomic level indicating that cell migration was significantly enriched in gene set enrichment analysis of mCherry positive cells (see below). However, due to the technical limitations mentioned above we did not validate this experimentally.

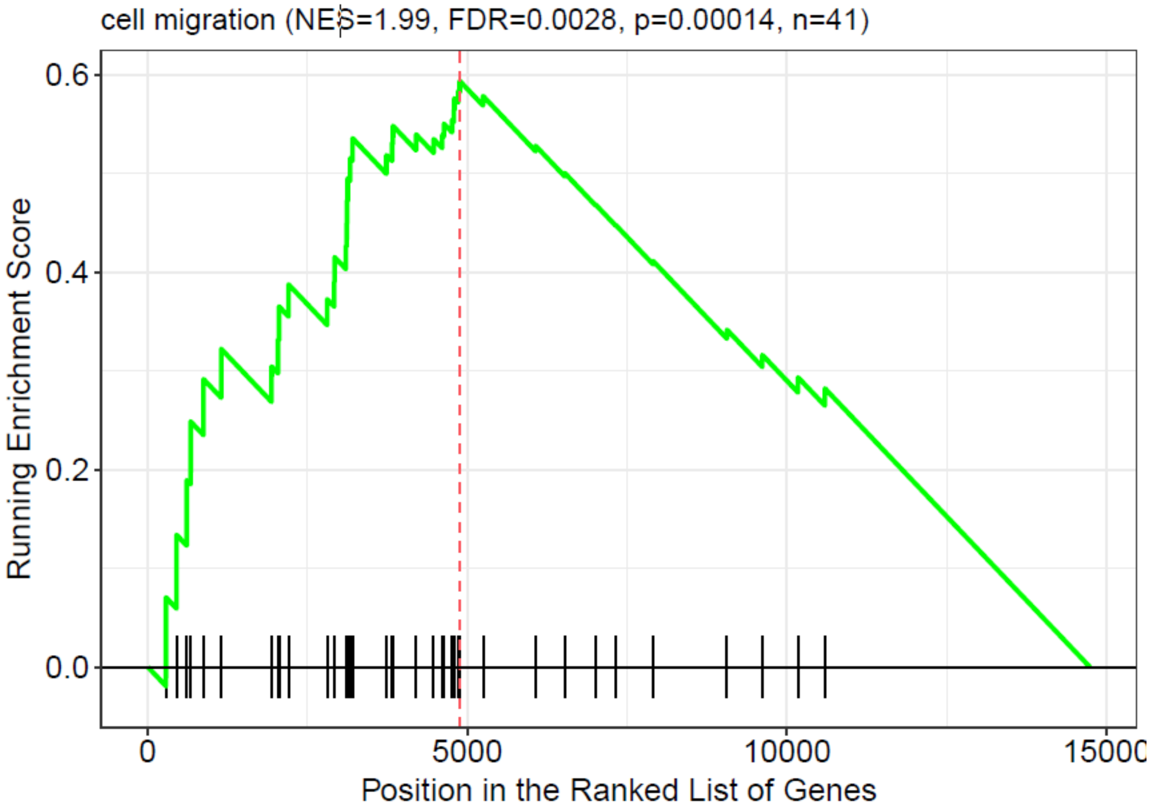


Specific points for consideration:

1. What is the fate of the increased number of RLRb+ planula cells after metamorphosis into the polyp? Given that this study identified a number of markers for these cells beyond RLRb, why were these cells not visualized in the adult polyp also?

As with several other *Nematostella* transgenic lines made by our lab as well as other labs by using meganuclease-assisted random genomic integration, the expression of the transgene is not stable throughout the life cycle. Unfortunately, in the case of the *RLRb::mCherry* line, we could not detect expression after metamorphosis, restricting the usefulness of this transgenic line to young life stages.

2. What are the RLRb negative phagocytic cells? Are they specialized also or have they just not yet upregulated RLRb?

Both options are plausible. Indeed, a previous work describing phagocytic cells in Hexacorallia described more than one morphology associated with phagocytic capacity [9]. In agreement, our morphological analysis of RLRb::mCherry FACS sorted cells, which is now included in the manuscript in Fig. S4, revealed that mCherry negative phagocytic cells were more heterogenous than mCherry positive. As suggested, cells with similar morphology (high granularity, large area) were included in both mCherry positive and negative cells, probably representing similar cells that did not express detectable mCherry levels at this particular time point.

3. Many of the gene names in the text seem vertebrate specific. Can the authors discuss issues of gene orthology?

As the reviewer correctly points out, we have used vertebrate (actually, mammalian) model species as a source of gene names for the *N. vectensis* gene models. We have added a new section in the methods detailing our methodology and data sources. We apologize for this omission in the previous version of the manuscript.

We think that using names transferred from model species where nomenclature is well-established and relatively stable offers advantages in terms of interpretability and readability of the manuscript, especially if reference genome and gene sets will change in the future (as it was recently the case in *N. vectensis* with the publication of a new chromosome-scale assembly). In any case, all *N. vectensis* gene identifiers used in the paper correspond to stable GeneBank entries (e.g. XM_048731786.1), which makes it very easy to retrieve the actual gene model and transcript/peptide sequence.

It is worth noting that our orthology inference approach is able to accommodate one-to-many, many-to-one or many-to-many orthology relationships between *N. vectensis* genes and the mammalian orthologs we used as a reference, which accounts for the complex evolutionary histories of gene families shared by cnidarians and mammalians. By doing so, we are able to assign names to ~60 % of *N. vectensis* genes. Furthermore, the tool we used to parse transcription factor gene trees and identify orthology relationships, *Possvm*, offers the possibility of extending gene name assignments to non-orthologous genes in a phylogeny-informed manner (e.g. to identify and label cnidarian-specific duplicates of a wider metazoan orthology group). This allowed us to assign a name to >98% of the TFs identified in *N. vectensis,* even when they lacked direct orthologs in mammals.

4. Page 10 Discussion end of Paragraph 1: There are actually a lot of studies of innate immunity in invertebrates outside of flies and C. elegans. Should broaden this statement or qualify the type of information that is limited.

We agree that numerous studies have examined innate immunity across diverse invertebrate lineages, including mollusks, annelids, cnidarians, and echinoderms. Our intention was to refer specifically to the **mechanistic and cell-type-resolved understanding of antiviral immunity**, which remains disproportionately based on work in *Drosophila* and *C. elegans*. We have revised the text to clarify that the limitation concerns the depth of mechanistic, cellular, and pathway-level information available across invertebrates, rather than innate immunity research in general. This clarification has now been incorporated into the Discussion.

5. Page 11, middle: Cells with macrophage- and neutrophil-like character may be ancient but it is unclear whether these represent ancient cell types or convergent cell phenotypes. Maybe qualify this statement.

We agree with the reviewer and modified the text accordingly. It now reads: "Phagocytic cells performing macrophage- or neutrophil-like functions are found across metazoans, including in sponges and tunicates, indicating that phagocytic immunity is ancient, although whether these cells share a common evolutionary origin or represent convergent solutions remains unclear [10,11]."

Minor issues:

Page 2 line 1 abstract: replace reconstructing with reconstruct

This has been corrected in the revised manuscript.

Page 33, legend, Fig.3I. Wrong reference number for Lewandowska?

This has been corrected in the revised manuscript.

Fig 5C: gene names are impossible to read. Maybe show in another way here and expand the volcano plot in the Supplementary figs?

This has been corrected in the revised manuscript. We enlarged the names of key immune genes and kept the figure in the main text.

Page numbers and line numbers would be useful in the review process.

These are now included in the revised manuscript.

**Reviewer #3 (Remarks to the Author):**

This manuscript addresses the evolutionary origin of immune cells by identifying and functionally characterizing a distinct population of cells activated by the viral mimic poly(I:C) in embryos of the cnidarian Nematostella vectensis. This study represents a substantial step forward and provides an absolute treasure trove of information and data on this topic that will help propel our understanding of how innate immunity evolved. The manuscript was a pleasure to read, well-organized and clear, and each section flowed logically from the previous one and the results were convincing. The approaches, including the innovative tools and creative experimental designs, are going to set a new standard for performing this type of study addressing similar questions. These tools include both standard flow cytometry and imaging flow cytometry, immunoprecipitation + mass spec for assessing antibody specificity, bulk RNAseq,

single-cell RNAseq, a transgenic reporter line, and a phagocytosis assay assessed by flow cytometry. Each of these approaches provides evidence and data to support the authors' ability to characterize the target cell population and support their hypothesis that this is a novel population of specialized immune cells involved in antiviral immunity. I appreciated the detail provided, including the gating strategies for all flow cytometry experiments.

I have two major critiques that must be addressed:

1. The authors make it clear throughout that they chose to focus on embryos of Nematostella, which is fine. They even provide some text in the discussion about how their results with embryos compare with results from previous studies by Cole et al. on adults and Margolis et al. on 4 week old polyps. However the authors need to provide a clear rationale for focusing on embryos. Was it simply that some of their approaches would only work well in embryos, or were there other reasons for this decision? Including a few more sentences on the rationale for choosing embryos and also a focus on the limitations of focusing on embryos (for making comparative claims) is warranted in the Introduction and again in the Discussion to tie it up.

We thank the reviewer for the constructive feedback. We totally agree with the comments and addressed them thoroughly in the revised manuscript.

Since we rely on microinjection for dsRNA delivery and there is currently no efficient method for delivering dsRNA into adult *Nematostella*, our experiments are limited to early developmental stages. This limitation is now clearly stated in the Introduction and Discussion.

1. Looking at the Data availability section, I feel what the authors have provided is very minimal, and woefully inadequate. If this is going to be a model or a new standard for how studies of novel cell types are characterized, as I hope it can be, then there is a huge amount of supplemental information/data that should be provided so others can replicate or follow what was done and validate the results or apply it to their own study. This is essential for others to be able to utilize the same approaches and analysis methods in other systems. I do appreciate the FigShare links that were provided, and I have looked through them. I also appreciate all of the supplemental figures & tables provided. However, what is missing is a supplemental methods text explanation of these supplemental figures & tables. For an example, please see Cazet et al. 2023 Genome Res. 33:

283-298. Supplemental_Material.pdf file for a guide for what needs to be included.

We agree with the reviewer and have expanded the description of each supplementary table to clarify how it was generated and what information it contains. These descriptions are provided in the Supplemental Material, following a format similar to the publication cited by the reviewer.

Also, it does seem that only minimal information has been provided for the single-cell RNAseq experiments and libraries. At a bare minimum, a table of statistics showing how many cells were loaded, how many were captured, and then the other summary statistics that are provided by CellRanger including fraction of reads per cell, mean reads per cell, median genes per cell, total genes detected, reads mapped to genome, et cet, must be provided. It is impossible to assess data quality without this information.

We agree with the reviewer that detailed quality control information is essential to assess single-cell RNA-seq data quality. We have therefore added a comprehensive table of CellRanger run statistics for both biological replicates in **Table S6 (sheet "QC_metrics", and CellRanger output available at DOI: 10.5281/zenodo.18390360; Nvec01_web_summary.html and Nvec02_web_summary.html)**, including the number of loaded and captured cells, fraction of reads in cells, sequencing depth, median genes and UMIs per cell, total genes detected, and genome and transcriptome mapping rates. These metrics demonstrate **high-quality libraries with excellent RNA capture and low ambient background**.

In addition, the **original Cell Ranger web summary reports** for both biological replicates are now provided in a dedicated public repository (Zenodo; DOI: **10.5281/zenodo.18390360**), allowing full inspection of all standard Cell Ranger QC metrics and visual summaries.

Finally, **Supplementary Fig. 5a,b** shows that the two replicates exhibit highly similar distributions across metacells and overlapping UMAP embeddings, indicating **consistently high data quality and no detectable batch effects**.

What also really needs to be provided in addition to these, is step-by-step descriptions of ALL computational analyses conducted as part of this study, including all relevant code, formatted both as markdown and HTML documents. This could be deposited in GitHub or a similar repository. For an example of a study where this was organized and executed quite well, again please see Cazet et al. 2023 Genome Res. 33: 283-298. Please follow this standard and provide similar descriptions of all computational analyses for each section of the manuscript.

We have reproduced all analyses and made all scripts and data-processing workflows used in this study publicly available to ensure full reproducibility. The GitHub repository

(https://github.com/ik-evoimmune/cnidarian_immune_cells) contains the original analysis scripts together with corresponding Markdown and rendered HTML reports that document each step of the workflow, including data preprocessing, quality control, feature extraction, statistical analyses, and data visualization.

Processed data files used as input for these analyses are deposited in a public repository (Zenodo; DOI: 10.5281/zenodo.18390360). Each analysis directory explicitly documents the source of its input data and the corresponding DOI. Large raw and processed datasets are hosted externally, while the GitHub repository provides all code and lightweight derived outputs required to reproduce the analyses and figures reported in the manuscript.

I also see that Table 1 shows the ImageStream raw data. This is not adequate in terms of flow cytometry data that needs to be provided. Please see the Current Protocols chapter "Preparing a Minimum Information about a Flow Cytometry Experiment (MIFlowCyt) Compliant Manuscript Using the International Society for Advancement of Cytometry (ISAC) FCS File Repository (FlowRepository.org)" https://currentprotocols.onlinelibrary.wiley.com/doi/full/10.1002/0471142956.cy1018s61

At a minimum, all of the raw .fcs files for ALL flow cytometry experiments must be deposited into the FlowRepository.

Since FlowRepository.org is temporarily unavailable, we have deposited the complete Imaging Flow Cytometry (ImageStream) dataset in a publicly accessible Zenodo repository (DOI: 10.5281/zenodo.18212341). The dataset is organized into separate raw and processed data archives to ensure full reproducibility. The *raw* archive contains all original .rif acquisition files from each experiment, while the *processed* archive contains the corresponding IDEAS output files (.cif, .daf), compensation matrices, analysis templates, and per-experiment feature exports. In addition, we provide an analysis_R folder containing the IDEAS-derived feature tables and a merged feature matrix used for downstream statistical analysis and PCA in R, together with detailed documentation of how these files were generated. This structure allows users to reproduce the complete analysis pipeline from raw ImageStream files through IDEAS processing to the final quantitative analyses presented in the manuscript.

In addition, we have deposited the complete raw flow cytometry dataset underlying this study in the same Zenodo repository. The repository contains all primary, unprocessed FCS files generated across the different assays described in the manuscript, including RLRb immunostaining, RLRb::mCherry reporter activation following poly(I:C) treatment, phagocytosis assays, apoptosis and proliferation measurements, and flow cytometry-based sorting for downstream fluorescence microscopy. Data are organized by assay, accompanied by detailed README files describing the experimental context and controls for each dataset.

Once all of this Data availability has been provided and updated, I will need to re-evaluate everything to make sure it was done to the necessary standard, that the data quality is high enough, and that the analyses were appropriate. As it stands, I am unable to evaluate the manuscript at this level. I have evaluated some other manuscripts that made an inadequate effort to provide code for all analyses in a study in a single text document for all experiments and it was completely impossible to follow & unacceptable for publication.

Beyond that, this study is a very impressive contribution and I personally look forward to further digging into the specific genes and pathways that were uncovered as there is a massive amount of cool science to follow up on. I also look forward to future studies performed in adults as that will be super interesting and an important next step to the story.

We thank the reviewer for their positive feedback and enthusiasm!

References


1 Cole, A. G. *et al.* Updated single cell reference atlas for the starlet anemone Nematostella vectensis. *Frontiers in Zoology* **21**, 8 (2024).

2 Margolis, S. R. *et al.* The cyclic dinucleotide 2′ 3′-cGAMP induces a broad antibacterial and antiviral response in the sea anemone Nematostella vectensis. *Proceedings of the National Academy of Sciences* **118**, e2109022118 (2021).

3 Iwama, R. E. & Moran, Y. Origins and diversification of animal innate immune responses against viral infections. *Nat Ecol Evol* **7**, 182-193 (2023). https://doi.org/10.1038/s41559-022-01951-4

4 Sheader, M., Suwailem, A. & Rowe, G. The anemone, Nematostella vectensis, in Britain: considerations for conservation management. *Aquatic Conservation: Marine and Freshwater Ecosystems* **7**, 13-25 (1997).

5 Hand, C. & Uhlinger, K. R. The unique, widely distributed, estuarine sea anemone, Nematostella vectensis Stephenson: a review, new facts, and questions. *Estuaries* **17**, 501-508 (1994).

6 Hand, C. & Uhlinger, K. R. The culture, sexual and asexual reproduction, and growth of the sea anemone Nematostella vectensis. *The Biological Bulletin* **182**, 169-176 (1992).

7 Kozlovski, I., Jaimes-Becerra, A., Aleshkina, D., Levy, M. & Moran, Y. Epigenomic and transcriptomic analyses reveal cnidocyte specialization in a sea anemone. *bioRxiv*, 2025.2010.2006.680633 (2025). https://doi.org/10.1101/2025.10.06.680633
8 Rabinowitz, C., Moiseeva, E. & Rinkevich, B. In vitro cultures of ectodermal monolayers from the model sea anemone Nematostella vectensis. *Cell and Tissue Research* **366**, 693-705 (2016).
9 Snyder, G. A. *et al.* Functional characterization of Hexacorallia phagocytic cells. *Frontiers in Immunology* **12**, 662803 (2021).
10 Nguyen, M. T., Liu, M. & Thomas, T. Ankyrin-repeat proteins from sponge symbionts modulate amoebal phagocytosis. *Molecular Ecology* **23**, 1635-1645 (2014).
11 Rosental, B. *et al.* Complex mammalian-like haematopoietic system found in a colonial chordate. *Nature* **564**, 425-429 (2018).
